# Supplementary material for: Migration of Dihydroxy Alkylamines and Their Possible Impurities from Packaging into Foods and Food Simulants: Analysis and Safety Evaluation
Source: Polymers (Basel). 2023 Jun 12;15(12):2656. doi: 10.3390/polym15122656 (PMC10303149; doi:10.3390/polym15122656)
Supplement: Supplementary file 1 [file polymers-15-02656-s001.zip › polymers-2451489-supplementary.pdf]

Polymers

Electronic Supplementary Material

Migration of dihydroxy alkylamines and its possible impurities from packaging into foods and food simulants: Analysis and safety evaluation

Antía Lestido-Cardama, Letricia Barbosa-Pereira, Raquel Sendón, Perfecto Paseiro Losada and Ana Rodríguez Bernaldo de Quirós

LC-MS/MS chromatograms of material samples, S4-S8 and S10-S12

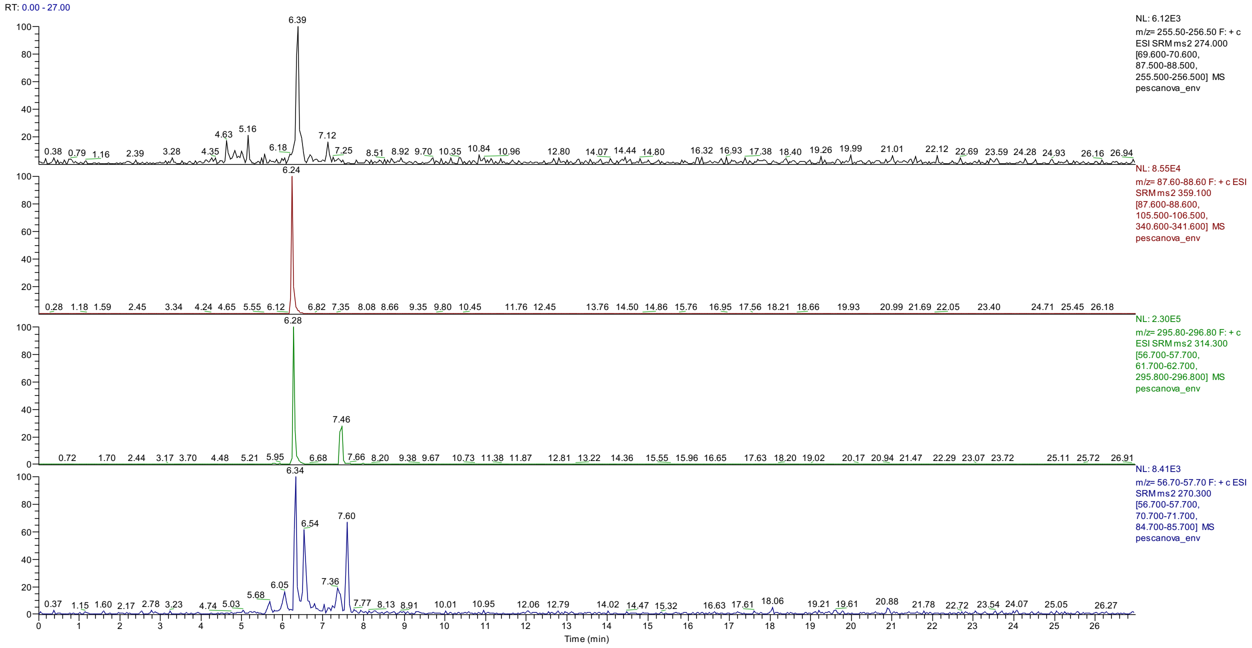

Figure S1.- LC-MS/MS chromatograms for ethanol extract of S4 packaging: (A) the transition for N,N-Bis(2-hydroxyethyl)dodecylamine (retention time: 5.16 min), (B) stearyldiethanolamine, (C) 2-(octadecylamino)ethanol and (D) octadecylamine.

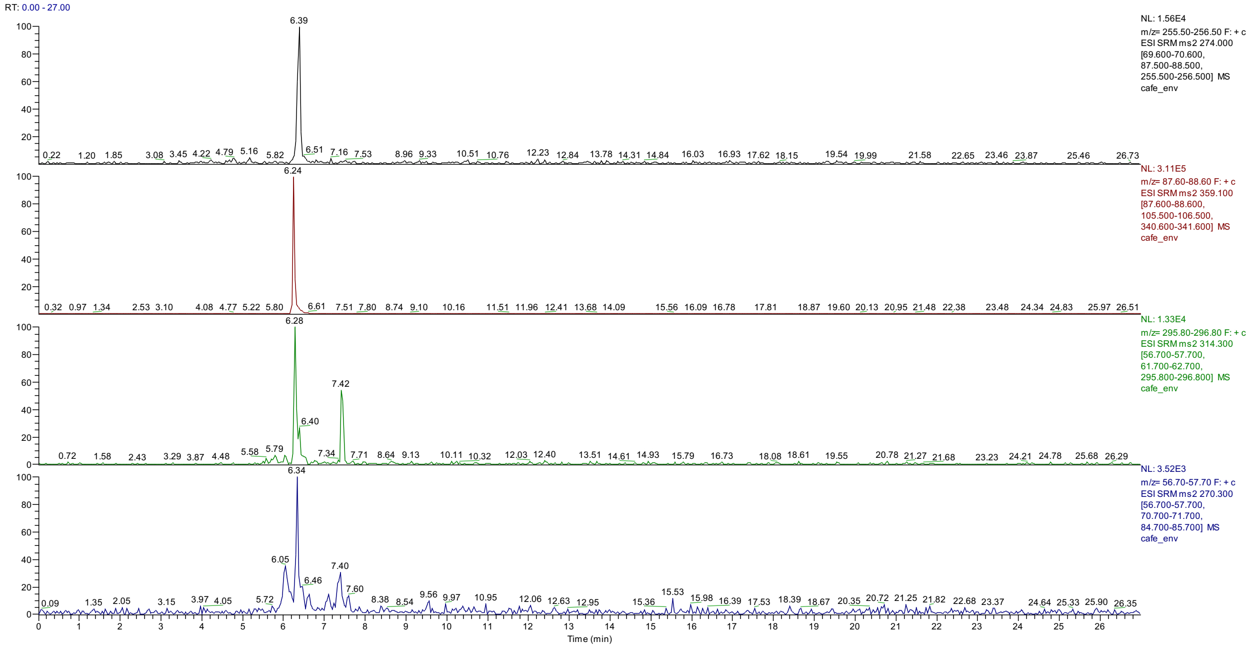

Figure S2.- LC-MS/MS chromatograms for ethanol extract of S5 packaging: (A) the transition for N,N-Bis(2-hydroxyethyl)dodecylamine (retention time: 5.16 min), (B) stearyldiethanolamine, (C) 2-(octadecylamino)ethanol and (D) octadecylamine.

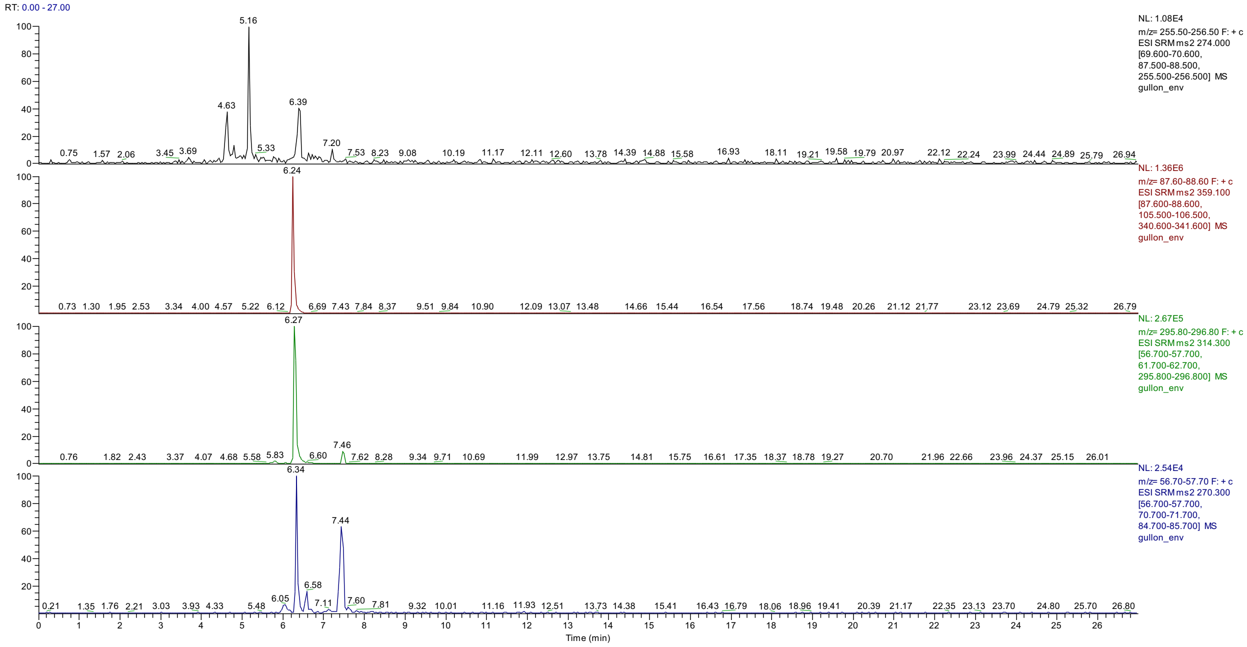

Figure S3.- LC-MS/MS chromatograms for ethanol extract of S6 packaging: (A) the transition for N,N-Bis(2-hydroxyethyl)dodecylamine (retention time: 5.16 min), (B) stearyldiethanolamine, (C) 2-(octadecylamino)ethanol and (D) octadecylamine.

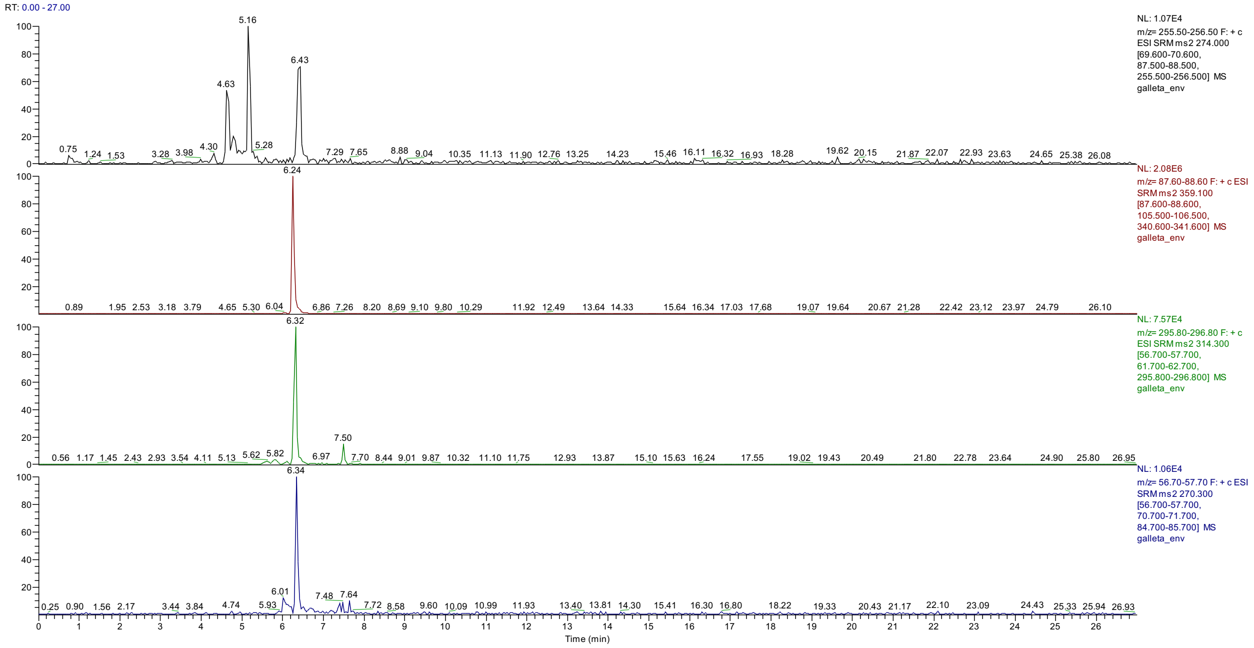

Figure S4.- LC-MS/MS chromatograms for ethanol extract of S7 packaging: (A) the transition for N,N-Bis(2-hydroxyethyl)dodecylamine (retention time: 5.16 min), (B) stearyldiethanolamine, (C) 2-(octadecylamino)ethanol and (D) octadecylamine.

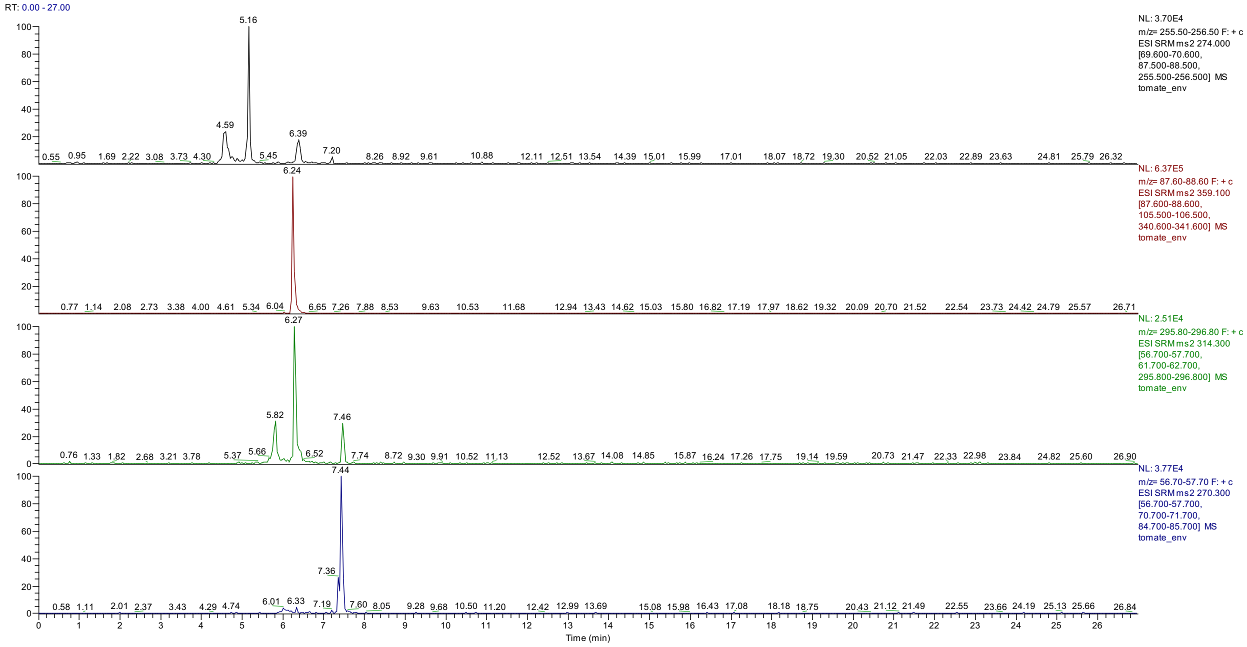

Figure S5.- LC-MS/MS chromatograms for ethanol extract of S8 packaging: (A) the transition for N,N-Bis(2-hydroxyethyl)dodecylamine (retention time: 5.16 min), (B) stearyldiethanolamine, (C) 2-(octadecylamino)ethanol and (D) octadecylamine.

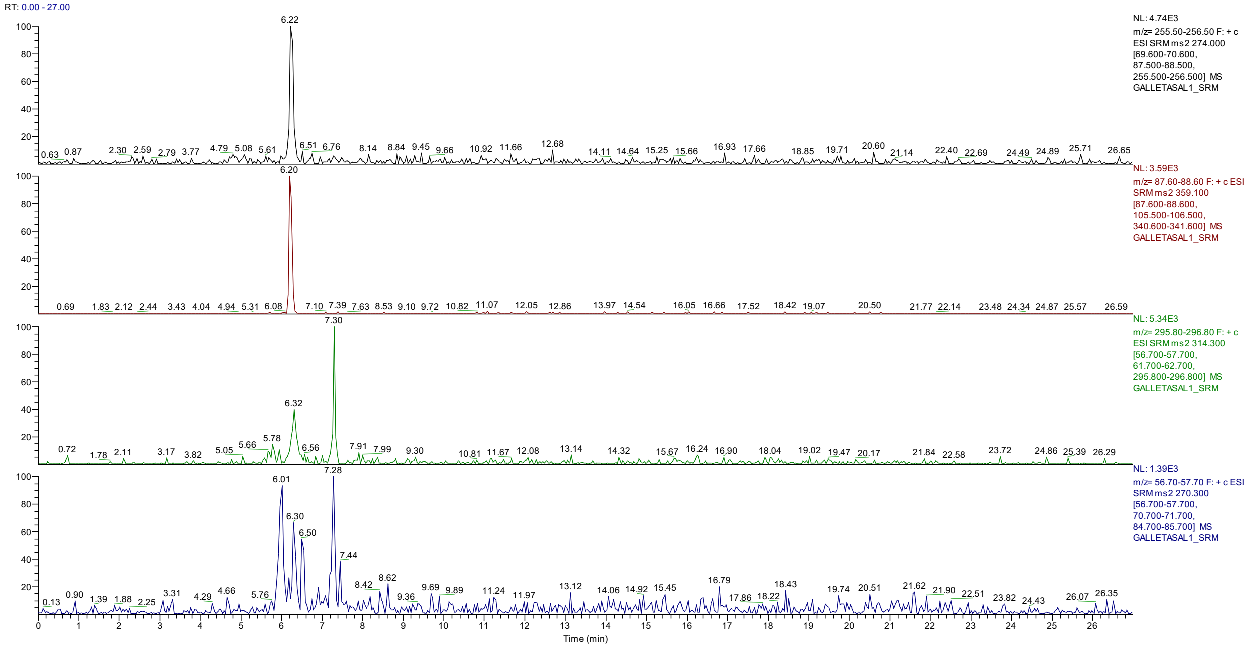

Figure S6.- LC-MS/MS chromatograms for ethanol extract of S11 packaging: (A) the transition for N,N-Bis(2-hydroxyethyl)dodecylamine (retention time: 5.16 min), (B) stearyldiethanolamine, (C) 2-(octadecylamino)ethanol and (D) octadecylamine.

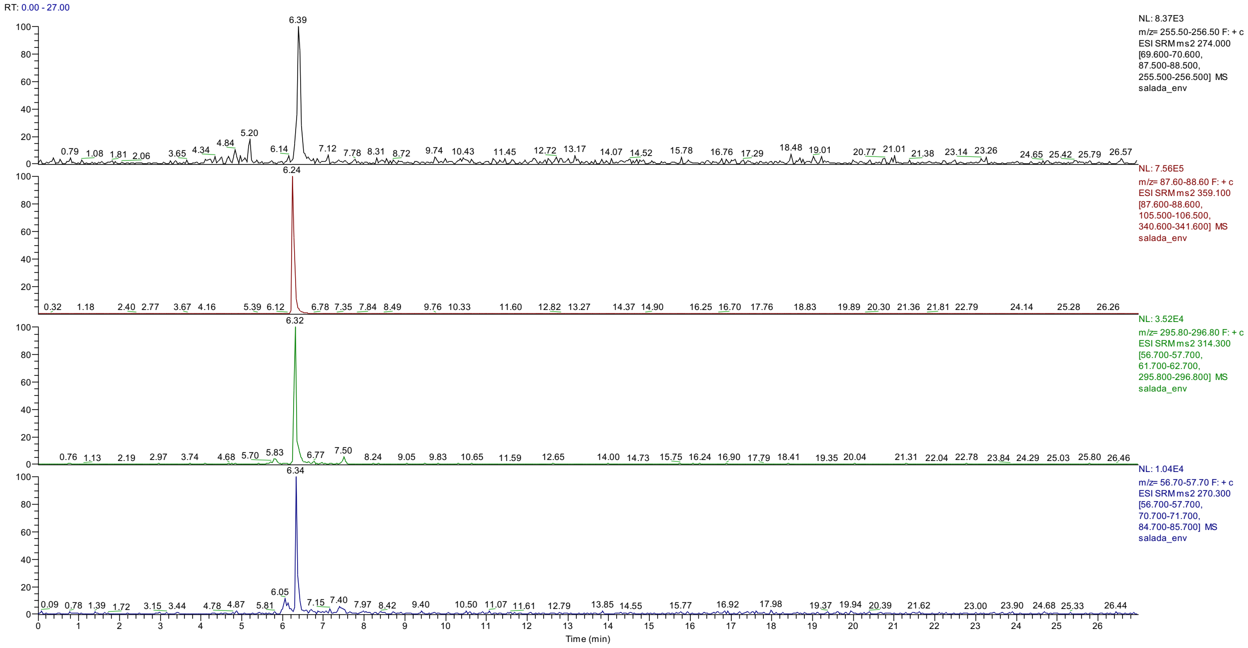

Figure S7.- LC-MS/MS chromatograms for ethanol extract of S12 packaging: (A) the transition for N,N-Bis(2-hydroxyethyl)dodecylamine (retention time: 5.16 min), (B) stearyldiethanolamine, (C) 2-(octadecylamino)ethanol and (D) octadecylamine.
